# Editorial Department

**Published:** 1884-01

**Authors:** 


### The Bistoury.

ELMIRA, N. Y., JANUARY, 1884.

The BISTOURY is published Quarterly, upon the first of April, July, October and January, at Fifty Cents a year in advance.

### Editorial Department.

A SMALL SUBSCRIPTION—Because the BISTOURY costs but fifty cents a year, many of our subscribers consider that that small sum is not worth the sending. Many have left their subscription accumulate until it amounts to several dollars, and then are mean enough to try to evade payment by ordering the journal stopped. Remember, you cannot stop any periodical until you have paid up in full.

THE INDEX—With this number is issued a full and complete index covering the past three years of the BISTOURY, commencing with April, 1881, and including the present number of January, 1884.

Physicians will find the index exceedingly valuable to them in permitting of easy reference to any ailment concerning which they desire hints for treatment. The household hints and recipes give abundant receipts for all manner of domestic employment. Examine, therefore, the index and learn of the great variety of topics upon which we undertake to inform you.

The BISTOURY will be sent to any address, substantially bound, containing all the numbers of past three years, fully indexed, for $2.50.

"A MERRY CHRISTMAS."—Everything points to the prospect of an unusually merry Christmas and a happy New Year. Merchants are doing a prosperous business, manufacturers have sold large quantities of their products; farmers have reaped bountiful crops; laborers have been steadily employed; we have had "good times" for the past year, and feel correspondingly good natured and disposed to take part in the holiday festivities. The shop windows are artistically arrayed with suitable gifts for everybody—for those of ample means, and for the people whose purses are not so plethoric. One can but wonder and admire the ingenuity of man in contriving devices that shall please the holiday purchasers. The display in all the departments of jewelry, fancy and toilet articles, dress and millinery goods, books, toys and the like, is simply wonderful and bewildering. Surely, Santa Claus will experience no difficulty in making his selections for his ponderous pack, this year.

The temptation to make presents in costliness, beyond one's ability, is great. Do not do it. Purchase within your means. A little memento—among friends—is often as acceptable as a more pretentious and costly offering. An expensive gift causes the one favored to feel under obligations, not wholly removed until one of equal value is returned. In that case, you purchase your gift and feel annoyed or distressed over the needless expenditure of money. Gift-giving is much abused, in latter days, among adults. The little people should be abundantly remembered. Their little hearts should be made glad over the advent of Santa Claus. In acquainting that mystical old gentleman of the desires of the little folks around your own hearthstone, forget not those of your washerwoman, or man-of-all-work, or of the poor folks who reside in the lane, close by. Be generous to your own children and also to those of the poor. Be frugal and wise in your purchases for adult relatives and friends. And so, let us all try to add to each other's enjoyment, so contributing to a "Merry Christmas and a Happy New Year!"

DANGER IN INHUMATION.—Of recent years, so much attention has been given, by medical men, to the investigation of the causes that lead to disease, that the origin of bacteria—now acknowledged to be responsible for much of the serious ailments of mankind—is becoming more and more familiar.

As the cities and towns grow larger and more numerous, the septic influences — the poison germs—are the better propagated, because of the more favorable opportunity for their culture. The more filthy and nasty the surroundings of the inhabiiants, the better are the conditions for breeding bacteria; therefore, the more prevalent becomes typhoid and other fevers, diphtheria, scarlet fever, and the like. Improper drainage and poor sewerage, where the refuse from the house is permitted to rot upon the surface of the ground, or, through the cesspools, finds its way into the wells, where millions of disease-producing bacteria are developed and introduced into the system, through the drinking water, are responsible for our septic diseases.

Instances are by no means lacking to abundantly prove these statements. Next to cesspools in bacteria-producing qualities, the graveyard stands foremost. Usually, these places for inhumation are selected upon some gravelly hill or knoll upon the outskirts of the city. This is the very best death-dealing situation that could possibly be desired. The rotting, putrid body is there invaded with the bacteria that live and thrive best in that sort of filth. The body is out of the reach of frost, the rains easily reach it, and wash the death-dealing germs through the porous soil to the streams and wells in the valley below. Then, the slimy, squirming worms, and hideous bugs that banquet upon the body and hold high carnival there, crawl to the surface, distributing their poisonous nastiness upon grass and flower, that innocent child or fond mother weeping there, may pluck the bright-eyed daisy, laden with its dreadful germs, and carry them home to poison the living.

Put it off as we may, the time *must* come when permission to endanger the living by burying the dead, will be prohibited not only by law, but also by the good sense of intelligent people. We long to see the day when the barbaric method of burying the body—a plan engendered, and having its origin in, the superstitious idea of the resurrection of the physical body—will be superceded by the cleanly, wholesome, beautiful method of cremation.

---

"BEFORE THE CLASS."—In our large cities are to be found one or more medical colleges that conduct clinics, where the poor people are permitted to appear and receive treatment for their various ailments, at the hands of noted professors in the several departments of medicine and surgery. When these clinics are properly conducted, the arrangement is mutually productive of good results. The patient, who is operated upon, is benefitted, and the student who looks on, is instructed. This system of receiving gratuitous treatment is known as "appearing before the class." Few people know how these clinics are conducted; and, for that reason, we see very many availing themselves of the opportunity afforded for free treatment, who are able to pay a physician, in private practice, a reasonable fee, and who would gladly do so, did they know to what they would have to submit in appearing in a public amphitheatre.

If these free clinics were wholly devoted to the really poor—those who are absolutely unable to pay for surgical or medical services—the benefits to both patient and student would be greatly enhanced. But, as now conducted, where the clinics are jammed with persons anxious to avail themselves of the services of skillful operators without cost, neither the service nor its results can be, from the very nature of the situation, satisfactory. The surgeon is overwhelmed with patients. He hurries them through the amphitheatre—operates upon them before thoroughly under the anæsthetic—does it hurriedly, sometimes awkwardly, and often carelessly. The patient is unnecessarily exposed. The operation is needlessly delayed, that any of the two or three hundred students who care to look at it, may do so as the table is whirled about. Every visiting physician who has been favored with admission to the amphitheatre, is invited to poke his finger into the wound, give the probe an extra jam or inspect the protruding viscera, all this, for the purposes of instruction. A young girl or an aged mother is placed upon the table to have her nude form exposed to a gaping crowd of young men, who comment coarsely upon the display. The surgeon draws his knife in a display-sort-of-way, over the tumor, stops to exhibit the incision to his class, comments upon the object to be attained, stops to show any important step of the procedure, tears out the tumor at last, holds it aloft to the admiring gaze and plaudits of the class; and, while the bystanders are fingering the wound, the assistants dressing it, the patient is groaning and the students guying each other. And that is a free clinic! It is a constant wonder to us that as good results are obtained as really prevail from this system of operating.

Surgeons are really to be congratulated for operating as well as they do, when so handicapped. Persons needing the services of surgeons, and who are able to pay *something*, should not present themselves at these clinics and so crowd out those who deserve the free services there to be had. Less supply of subjects will tend to make less hurried, and therefore, more careful operating. There is probably no surgeon of any eminence but that will give his services for a sum within the ability of the patient to pay. Apply to him at his private office, but avoid "going before the class," unless you desire to be subjected to a process that having once seen, you would never, under any circumstances, undergo again.

